# Advancements in the Synthesis and Functionalization of Zinc Oxide-Based Nanomaterials for Enhanced Oral Cancer Therapy

**DOI:** 10.3390/molecules29112706

**Published:** 2024-06-06

**Authors:** Jinjin Pei, Prabhu Manickam Natarajan, Vidhya Rekha Umapathy, Bhuminathan Swamikannu, Nandini Manickam Sivaraman, Lakshmi Krishnasamy, Chella Perumal Palanisamy

**Affiliations:** 1 Qinba State Key Laboratory of Biological Resources and Ecological Environment, 2011 QinLing-Bashan Mountains Bioresources Comprehensive Development C. I. C., Shaanxi Province Key Laboratory of Bio-Resources, College of Bioscience and Bioengineering, Shaanxi University of Technology, Hanzhong 723001, China; xnjinjinpei@163.com; 2 Department of Clinical Sciences, d Centre of Medical and Bio-Allied Health Sciences and Research, College of Dentistry, Ajman University, Ajman P.O. Box 346, United Arab Emirates; 3 Department of Public Health Dentistry, Thai Moogambigai Dental College and Hospital, Chennai 600 107, Tamil Nadu, India; drvidhyarekha@gmail.com; 4 Department of Prosthodontics, Sree Balaji Dental College and Hospital, Pallikaranai, Chennai 600 100, Tamil Nadu, India; bhumi.sbdch@gmail.com; 5 Department of Microbiology, Sree Balaji Medical College and Hospital, Bharath University, Chennai 600 100, Tamil Nadu, India; drnandini@bharathuniv.ac.in (N.M.S.); laksh45@gmail.com (L.K.); 6 Department of Chemical Technology, Faculty of Science, Chulalongkorn University, Bangkok 10330, Thailand

**Keywords:** ZnO nanomaterials, synthesis methods, surface functionalization, oral cancer

## Abstract

The fabrication of zinc oxide-based nanomaterials (including natural and synthetic polymers like sulfated polysaccharide, chitosan, and polymethyl methacrylate) has potential to improve oral cancer treatment strategies. This comprehensive review explores the diverse synthesis methods employed to fabricate zinc oxide nanomaterials tailored for oral cancer applications. Several synthesis processes, particularly sol–gel, hydrothermal, and chemical vapor deposition approaches, are thoroughly studied, highlighting their advantages and limitations. The review also examines how synthesis parameters, such as precursor selection, the reaction temperature, and growth conditions, influence both the physicochemical attributes and biological efficacy of the resulting nanomaterials. Furthermore, recent advancements in surface functionalization and modification strategies targeted at improving the targeting specificity and pharmaceutical effectiveness of zinc oxide-based nanomaterials in oral cancer therapy are elucidated. Additionally, the review provides insights into the existing issues and prospective views in the field, emphasizing the need for further research to optimize synthesis methodologies and elucidate the mechanisms underlying the efficacy of zinc oxide-based nanoparticles in oral cancer therapy.

## 1. Introduction

Oral cancer remains a significant public health concern globally, with its incidence steadily rising in recent years [1]. Despite breakthroughs in treatment modalities such as surgery, chemotherapy, and radiotherapy, the long-term outlook for individuals with oral cancer remains poor, owing mostly to late-stage detection and the limited efficacy of available therapies against aggressive tumor morphologies [2]. As a result, there is a critical requirement to develop unique therapeutic techniques that can selectively target oral cancer cells while limiting systemic toxicity [3]. In this context, nanotechnology has become known as an exciting approach for enhancing cancer diagnosis and treatment [4]. Nanomaterials, with their unique physicochemical properties and tunable surface characteristics, offer versatile platforms for drug delivery, imaging, and targeted therapy [5,6]. Zinc oxide (ZnO)-based nanomaterials have gained popularity in recent years due to their biocompatibility, low toxicity, and photocatalytic characteristics. Furthermore, ZnO nanoparticles have inherent anticancer activities due to their production of reactive oxygen species (ROS) when exposed to light or UV radiation, making them promising candidates for cancer therapy [7].

The manufacturing of ZnO-based nanomaterials is crucial for their use in oral cancer therapy. Several synthesis methods have been established to produce ZnO nanoparticles with exact sizes, shapes, and surface characteristics [8]. Sol–gel, hydrothermal synthesis, chemical vapor deposition, and other novel procedures are among the options, each with its own set of benefits and drawbacks. Understanding the impact of synthesis factors such as precursor selection, reaction temperature, and growth conditions is critical for adapting the properties of ZnO nanoparticles to the specific needs of oral cancer applications [9]. Furthermore, surface functionalization and modification strategies play an essential role in enhancing the targeting specificity and therapeutic efficacy of ZnO-based nanomaterials. By conjugating targeting ligands or enclosing therapeutic agents within the structure of ZnO nanoparticles, researchers can achieve selective accumulations within tumor tissues and improve drug delivery efficiency while minimizing off-target effects. Moreover, surface modifications can also enhance the stability and biocompatibility of ZnO nanomaterials, facilitating their translation into clinical practice [10].

Despite the significant progress made in the field of ZnO-based nanomaterials for oral cancer applications, several challenges still need to be addressed. These include optimizing synthesis methodologies to improve reproducibility and scalability, elucidating the mechanisms underlying the anticancer activity of ZnO nanoparticles, and addressing concerns regarding their long-term biocompatibility and safety [11]. Additionally, the development of robust preclinical models that accurately recapitulate the complex tumor microenvironment of oral cancer is essential for evaluating the efficacy and safety of ZnO nanomaterials in vivo [12]. This comprehensive analysis aims to provide a thorough understanding of the synthesis of ZnO-based nanomaterials for oral cancer applications.. Various synthesis methods are used, the impact of synthesis parameters on nanomaterial properties, and the importance of surface functionalization in improving their therapeutic efficacy are analyzed. Furthermore, this review discussed current achievements in the field, as well as the challenges and future prospects for developing ZnO-based nanomaterials as effective oral cancer therapies. This review aims to enhance nanotechnology-based approaches for precise cancer therapy by integrating existing knowledge and recommending topics for future research.

## 2. Synthesis Methods for Zinc Oxide-Based Nanomaterials

**Figure 1:**
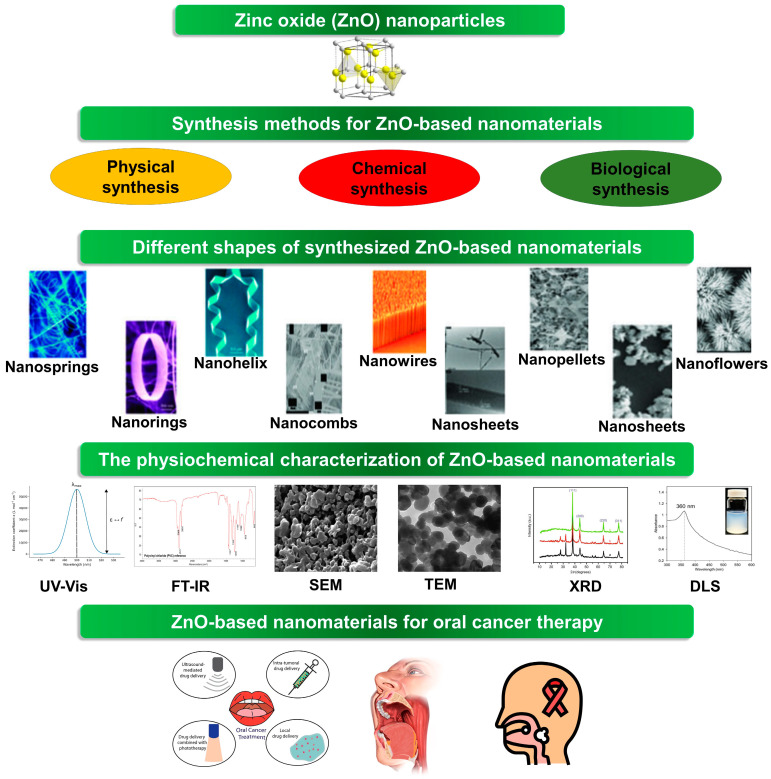
The fabrication of ZnO-based nanomaterials for oral cancer therapy.

Nanotechnology has revolutionized the field of materials science, offering precise control over the synthesis of nanomaterials with tailored properties for various applications, including cancer therapy [13]. In the context of oral cancer treatment, ZnO-based nanomaterials have garnered significant interest due to their inherent anticancer properties and biocompatibility. Numerous synthesis approaches have been established to produce ZnO nanoparticles with exact sizes, shapes, and surface properties [14]. This section delves into some of the most generally used synthesis methods for ZnO-based nanomaterials (Figure 1).

### 2.1. Physical Synthesis Methods

Physical methods for the synthesis of zinc oxide-based nanomaterials rely on physical processes such as vapor deposition, laser ablation, and mechanical milling to fabricate nanostructures [15]. A list of some common physical synthesis methods includes: Chemical Vapor Deposition (CVD): In CVD, ZnO nanostructures are grown through the reaction of precursor vapors on a substrate at elevated temperatures. This method offers precise control over film thickness, morphology, and crystallinity [16]. Physical Vapor Deposition (PVD)*:* PVD methods, such as sputtering and evaporation, are employed to physically deposit ZnO thin films or nanostructures onto substrates without involving chemical reactions [17]. Pulsed Laser Deposition (PLD): PLD involves the ablation of a ZnO target made by a pulsed laser in a vacuum or controlled atmosphere, resulting in the deposition of ZnO thin films or nanostructures on a substrate [18]. Electrodeposition: Electrodeposition is a physical method in which ZnO nanostructures are grown via electrodeposition from an aqueous solution containing zinc ions onto a conductive substrate under the influence of an electric field [19]. Molecular Beam Epitaxy (MBE): MBE is a highly controlled physical vapor deposition technique used to grow epitaxial ZnO thin films layer by layer on a substrate under ultra-high vacuum conditions [20]. Sputtering: Sputtering represents a physical vapor deposition method where energetic ions bombard a ZnO target, resulting in the ejection and deposition of atoms onto a substrate, thereby forming thin films or nanostructures [21]. Evaporation: Evaporation involves the heating of a ZnO source material in a vacuum chamber, causing it to evaporate and condense onto a substrate to form thin films or nanostructures [22]. Magnetron Sputtering: Magnetron sputtering is a variation of sputtering in which a magnetic field is applied to increase the efficiency of the ionization and deposition of ZnO thin films or nanostructures [21]. Physical Mixing and Milling: Physical mixing and milling methods entail mechanically grinding or milling bulk ZnO materials to diminish their size to a nanoscale. This approach proves fitting for generating ZnO nanoparticles or nanocomposites [23]. Gas Phase Synthesis: Gas phase synthesis methods such as aerosol techniques and gas-phase condensation involve the nucleation and growth of ZnO nanoparticles from vapor-phase precursors in a controlled gas environment [24]. These physical synthesis methods offer precise control over the size, morphology, and crystallinity of the resulting ZnO nanostructures, making them suitable for various applications in electronics, optoelectronics, catalysis, and sensing.

### 2.2. Chemical Synthesis Methods 

Chemical methods for the synthesis of zinc oxide-based nanomaterials involve chemical reactions between precursor compounds to form ZnO nanostructures [15]. A list of some common chemical synthesis methods are briefly described in this section. The Sol–Gel Method: This process entails the hydrolysis and condensation of metal alkoxides or salts within a solution, leading to the formation of a sol, which subsequently undergoes gelation and drying processes to obtain ZnO nanoparticles [25]. Hydrothermal/Solvothermal Synthesis: ZnO nanostructures are created within high-temperature and high-pressure environments using aqueous or organic solvents. This method facilitates precise control over the synthesis, yielding diverse nanostructures like nanorods, nanowires, and nanosheets [26]. Precipitation Method: Zinc salts (e.g., zinc acetate, zinc nitrate) are precipitated in the presence of a base (e.g., sodium hydroxide, ammonium hydroxide) or a complexing agent, leading to the formation of ZnO nanoparticles [27]. Microemulsion Method: This involves the formation of nanoscale droplets (microemulsions) containing precursor compounds, followed by the reaction and nucleation of ZnO nanoparticles within these droplets [28]. Combustion Synthesis: ZnO nanoparticles are synthesized by the combustion of suitable precursors such as zinc nitrate mixed with a fuel source (e.g., urea, glycine), resulting in highly crystalline ZnO nanoparticles [29]. Template-Assisted Methods: Templates such as porous membranes, polymers, or self-assembled monolayers are used to control the size and shape of ZnO nanostructures during synthesis, including electrodeposition, electrospinning, and layer-by-layer assembly [30]. Microwave-Assisted Synthesis: Microwave irradiation promotes rapid heating and uniform nucleation, leading to reduced reaction times and improved crystallinity during the synthesis of ZnO nanostructures [31]. Complex Precursor Decomposition: Complex precursors containing Zn and organic ligands are decomposed thermally or hydrothermally to yield ZnO nanoparticles with controlled sizes and morphologies [32]. Spray Pyrolysis: ZnO nanoparticles are synthesized by the thermal decomposition of precursor solutions sprayed onto a heated substrate, allowing for the large-scale production of ZnO thin films or nanostructures [33]. These chemical synthesis methods offer versatility in controlling the size, shape, morphology, and properties of ZnO nanomaterials, making them suitable for various applications in electronics, optoelectronics, catalysis, sensors, and biomedical devices [34].

### 2.3. Biological Synthesis Methods

Biological approaches to synthesizing zinc oxide-based nanomaterials utilize biological agents, including plant extracts, microorganisms, or enzymes, serving as both reducing and stabilizing agents [35]. The list of some common biological synthesis methods includes: Plant-Mediated Synthesis: Plant extracts harboring phytochemicals like flavonoids, phenols, and terpenoids serve as both reducing and capping agents in the synthesis of ZnO nanoparticles. Various plant species including Aloe vera, Azadirachta indica (neem), and green tea have been utilized for this purpose [36]. Microbial Synthesis: Microorganisms such as bacteria, fungi, and yeast are employed to biosynthesize ZnO nanoparticles through the reduction of zinc salts in culture media. Microbial enzymes and metabolites play a crucial role in the reduction and stabilization of ZnO nanoparticles [37]. Algal-Mediated Synthesis: Algae are utilized for the green synthesis of ZnO nanoparticles due to their ability to secrete bioactive compounds and enzymes. Algal extracts or biomasses are used as reducing agents to convert zinc salts into ZnO nanoparticles [38]. Enzyme-Assisted Synthesis: Enzymes such as urease, alkaline phosphatase, and lysozyme are used to facilitate the biogenic synthesis of ZnO nanoparticles by catalyzing the reduction of zinc ions into ZnO in aqueous solutions [39]. Cell-Free Extracts: Cell-free extracts obtained from plants, microorganisms, or algae are used as bioreducing agents for the synthesis of ZnO nanoparticles. These extracts contain a rich mixture of biomolecules such as proteins, carbohydrates, and secondary metabolites that aid in nanoparticle formation [40]. Biogenic Precipitation: Biogenic precipitation involves the formation of ZnO nanoparticles through the biomineralization process mediated by biological entities. This method mimics the natural mineralization processes observed in organisms such as diatoms and mollusks [41]. Extracellular Synthesis: ZnO nanoparticles are synthesized extracellularly by microorganisms or plant cells, where the reduction and nucleation of zinc ions occurs outside the cell in the presence of biomolecules released by biological entities [42]. Intracellular Synthesis: ZnO nanoparticles are synthesized intracellularly within microbial or plant cells, where zinc ions are transported into the cell and reduced into ZnO nanoparticles in specialized organelles or compartments [43]. Biocomposite Formation: Biocomposite materials containing ZnO nanoparticles embedded within a biopolymeric matrix are synthesized using biological entities as reducing and stabilizing agents. These biocomposites exhibit enhanced properties for various applications [44]. Green Synthesis Approaches: Biological methods for ZnO nanoparticle synthesis are considered to be green and eco-friendly alternatives to conventional chemical methods, offering low toxicity, biocompatibility, and sustainability [45]. These biological synthesis methods offer advantages such as environmentally friendly synthesis routes, mild reaction conditions, and the potential for the large-scale production of ZnO nanoparticles for various applications including uses in biomedical, agricultural, and environmental sectors [46].

A wide range of synthesis methods are available for the fabrication of ZnO-based nanomaterials with tailored properties for various applications. Each method offers unique advantages and limitations (Table 1), and the choice of technique depends on factors such as the desired nanostructure morphology, scalability, and targeted applications (Table 2) [47]. Advances in synthesis techniques continue to drive the development of ZnO nanomaterials with enhanced performances and functionalities, paving the way for their widespread adoption in diverse technological fields [34].

## 3. Influence of Synthesis Parameters on Nanomaterial Properties

The properties of ZnO-based nanomaterials crucially depend on various synthesis parameters, including precursor selection, reaction temperature, and growth conditions. By finely adjusting these parameters, it becomes possible to exert precise control over the nanoparticle size, shape, crystallinity, surface morphology, and surface chemistry. These factors subsequently impact the physicochemical properties and the biological behavior of the nanomaterials [48]. In this section, how these synthesis parameters influence the properties of ZnO-based nanomaterials specifically tailored for oral cancer applications is discussed.

### 3.1. Precursor Selection

The selection of a precursor is pivotal in shaping the chemical composition, purity, and crystallinity of ZnO-based nanomaterials. Typical precursors utilized for ZnO synthesis encompass zinc salts like zinc acetate, zinc nitrate, zinc chloride, and zinc sulfate, alongside ZnO nanoparticles or complexes [49]. The chemical composition and solubility of the precursor in the reaction medium significantly influences the nucleation and growth kinetics of ZnO nanoparticles. Zinc acetate, for example, is a commonly used precursor due to its high solubility in polar solvents and its ability to form homogeneous precursor solutions. On the other hand, zinc chloride is preferred for nonaqueous synthesis routes due to its high volatility and low solubility in water [50]. Additionally, the concentration of the precursor affects the rate of nucleation, the distribution of particle sizes, and the morphology of the ZnO nanoparticles. Higher precursor concentrations typically result in higher nucleation rates and smaller particle sizes due to the increased availability of zinc ions for nucleation and growth. However, excessively high precursor concentrations can lead to particle aggregation and Ostwald ripening, resulting in broad size distributions and irregular morphologies [51]. In addition to the choice of precursor, the presence of dopant ions or additives in the precursor solution can modify the properties of ZnO nanoparticles. Dopant ions, such as aluminum, indium, or gallium, can alter the bandgap, optical properties, and photocatalytic activity of the ZnO nanomaterials, making them suitable for specific applications, such as photodynamic therapy or imaging [52].

In physical synthesis methods such as Chemical Vapor Deposition (CVD) and Physical Vapor Deposition (PVD), a low precursor concentration can result in slower deposition rates, leading to thinner films and incomplete coverage, which negatively impacts the uniformity and crystallinity of ZnO films. Similarly, in electrodeposition, an insufficient precursor concentration can lead to lower nucleation rates, resulting in sparse and uneven nanostructure growth that compromises the film’s density and uniformity. In Pulsed Laser Deposition (PLD), a reduced precursor concentration can decrease the ablation efficiency, leading to thinner films with potential defects and lower crystallinity [53].

In chemical synthesis methods, the effects of low precursor concentration are equally significant. In the sol–gel process, this can lead to incomplete hydrolysis and condensation, resulting in smaller and less-uniform nanoparticles, and a lower viscosity sol that affects the gelation process. In hydrothermal or solvothermal synthesis, lower precursor concentration slows nucleation and growth rates, producing smaller, less crystalline particles and potentially irregular shapes. The precipitation method can suffer from lower yields and a broader size distribution due to incomplete reactions. Microwave-assisted synthesis may experience reduced heating efficiency and slower reaction rates, affecting the size and uniformity of the nanoparticles produced [54].

Biological synthesis methods are also impacted by the precursor concentration. In plant-mediated synthesis, a low precursor concentration can lead to insufficient interactions between plant extracts and zinc ions, resulting in lower yields and smaller, less-uniform nanoparticles. Microbial synthesis can be limited by the availability of zinc ions for microbial reduction, leading to slower synthesis rates and lower yields. In enzyme-assisted synthesis, a low precursor concentration can result in an incomplete reduction of zinc ions, yielding smaller and less-crystalline nanoparticles, with enzymes potentially becoming less efficient [55].

Other factors such as temperature, pH, reaction time, surfactants, and solvents also significantly impact ZnO synthesis. Higher temperatures generally enhance reaction rates and crystallinity but can lead to larger particle sizes and potential agglomeration, while lower temperatures yield smaller particles with better size distribution control [48]. The pH of the reaction medium affects the solubility of the zinc precursors and the stability of the ZnO nanoparticles, with alkaline conditions favoring ZnO formation. Extended reaction times can improve crystallinity but may cause agglomeration and the loss of nanoscale properties. Surfactants and stabilizers help control particle size and shape by preventing agglomeration and guiding growth, though they must be optimized to avoid introducing impurities [51]. The choice of solvent influences the solubility of precursors, reaction kinetics, and the morphology of the ZnO nanostructures, with aqueous solvents being more environmentally friendly and organic solvents offering better control over particle size and shape [56].

The concentration of precursors is a crucial parameter in ZnO synthesis, influencing the physical and chemical properties of the nanomaterials [57]. Low precursor concentrations can lead to incomplete reactions, lower yields, and suboptimal nanostructure characteristics. Addressing the precursor concentration alongside factors like temperature, pH, reaction time, surfactants, and solvents provides the comprehensive understanding necessary for optimizing ZnO synthesis for various applications. By systematically studying these parameters, researchers can tailor the synthesis process to produce ZnO nanomaterials with the desired properties and functionalities [25].

### 3.2. Reaction Temperature

The reaction temperature is a critical parameter that significantly influences the nucleation, growth kinetics, phase composition, and crystallinity of ZnO nanoparticles. Elevated reaction temperatures typically accelerate nucleation and growth rates, resulting in smaller particle sizes and enhanced crystallinity. Nonetheless, excessively high temperatures may also trigger thermally induced phase transformations, such as the formation of metastable phases or the aggregation of nanoparticles [57]. For example, in the hydrothermal synthesis of ZnO nanowires, higher reaction temperatures promote the anisotropic growth of nanowires along specific crystallographic directions, resulting in longer and more uniform nanostructures. Conversely, lower reaction temperatures may favor the formation of nanoparticles with different morphologies, such as nanospheres or nanoplates [58]. Moreover, the reaction temperature can influence the degree of crystallinity and the defects in the ZnO nanoparticles, which in turn affect their optical, electronic, and photocatalytic properties. Higher reaction temperatures promote the formation of well-crystallized ZnO nanoparticles with fewer defects, resulting in enhanced photocatalytic activity and stability [25]. Optimizing the reaction temperature is crucial for controlling the size, morphology, and properties of ZnO nanoparticles for oral cancer applications. By carefully adjusting the reaction temperature to be within a suitable range, researchers can tailor the properties of ZnO nanomaterials to meet the specific requirements of targeted therapy, drug delivery, or imaging in oral cancer treatment [57].

### 3.3. Growth Conditions

The growth conditions, including the pH, solvent composition, reaction time, and precursor-to-solvent ratio, also play a critical role in determining the properties of ZnO-based nanomaterials. These parameters influence the kinetics of nucleation, growth, and crystal growth, as well as the stability and dispersibility of nanoparticles in the reaction medium [59]. The pH level of the reaction solution impacts the solubility of the zinc ions and the hydrolysis rate of the precursor species, consequently influencing the nucleation and growth kinetics of the ZnO nanoparticles. Generally, alkaline pH conditions accelerate nucleation and growth rates by promoting the hydrolysis of zinc precursor species and the formation of stable nuclei. However, excessively high pH values may result in the generation of undesirable byproducts or phase impurities, such as zinc hydroxides or zinc carbonates [60]. The choice of solvent and solvent composition also influences the solubility of the precursor species, the stability of the reaction intermediates, and the morphology of the ZnO nanoparticles. For the sol–gel synthesis of ZnO nanoparticles, polar solvents like water or alcohols are frequently employed due to their ability to dissolve zinc salts and stabilize precursor solutions. Nonpolar solvents, such as hexane or toluene, may be employed for the synthesis of ZnO nanoparticles via nonaqueous routes, offering advantages such as better control over particle size and reduced agglomeration [61].

Furthermore, the reaction time and precursor-to-solvent ratio dictate the extent of particle growth and aggregation during the synthesis process. Extended reaction durations typically yield larger particle sizes and wider size distributions because of ongoing nucleation and Ostwald ripening. In contrast, shorter reaction times often produce smaller nanoparticles with more uniform morphologies [62]. Through the careful optimization of growth conditions, researchers can customize the size, morphology, crystallinity, and surface characteristics of ZnO nanoparticles to suit particular applications in oral cancer therapy. Fine-tuning these parameters allows for the development of ZnO-based nanomaterials with enhanced therapeutic efficacy, biocompatibility, and targeting specificity, paving the way for precise cancer treatment strategies [63].

## 4. Surface Functionalization and Modification Strategies

Surface functionalization and modification are essential for customizing the properties of ZnO-based nanomaterials for targeted oral cancer therapy [64]. By modifying the surface chemistry of ZnO nanoparticles, researchers can enhance their biocompatibility, stability, dispersibility, and targeting specificity, consequently, enhancing their therapeutic effectiveness while minimizing off-target effects [10]. In this section, various surface functionalization and modification strategies employed to optimize ZnO nanomaterials for oral cancer applications are explored.

### 4.1. Ligand Conjugation

Attaching targeting ligands, including antibodies, peptides, or small molecules, onto the surface of ZnO nanoparticles enables specific recognition and binding to cancer cells overexpressing corresponding receptors or biomarkers [65]. This targeted delivery approach enhances the accumulation of ZnO nanoparticles within the tumor microenvironment while reducing the nonspecific uptake by healthy tissues, thereby improving therapeutic outcomes and minimizing side effects [8]. An example includes the development of folate receptor-targeted ZnO nanoparticles designed for delivering anticancer drugs selectively to oral cancer cells that overexpress folate receptors. Folic acid, serving as a high-affinity ligand for folate receptors, can be conjugated onto the surface of ZnO nanoparticles via covalent coupling or surface adsorption methods. Upon systemic administration, folate-functionalized ZnO nanoparticles preferentially accumulate within folate receptor-positive oral cancer cells, leading to the enhanced cellular uptake and cytotoxicity of the encapsulated drugs [66]. Similarly, epidermal growth factor receptor (EGFR)-targeted ZnO nanoparticles have been explored for the precise targeting of EGFR-overexpressing oral cancer cells. Epidermal growth factor (EGF) or anti-EGFR antibodies can be conjugated onto the surface of ZnO nanoparticles to facilitate receptor-mediated endocytosis and intracellular drug delivery. This targeted approach enables the precise localization of therapeutic agents within the tumor tissue, enhancing their therapeutic efficacy while minimizing systemic toxicity [67].

### 4.2. Polymer Coating

Polymer coating is another effective strategy for the surface modification of ZnO nanoparticles, offering advantages like enhanced stability, biocompatibility, and drug-loading capacity [68]. Biocompatible polymers, such as polyethylene glycol (PEG), polyvinylpyrrolidone (PVP), or chitosan, can be adsorbed or covalently attached onto the surface of ZnO nanoparticles to form a stable coating layer [69]. PEGylation, in particular, has emerged as a widely used surface modification strategy for ZnO nanoparticles due to its ability to impart stealth properties and prolong the circulation time in vivo [70]. ZnO nanoparticles coated with PEG show decreased protein adsorption, opsonization, and clearance via the reticuloendothelial system (RES), resulting in extended systemic circulation and improved tumor accumulation through the enhanced permeability and retention (EPR) effect [71]. Furthermore, polymer coatings can serve as carriers for the therapeutic compounds that are encapsulated and released under controlled conditions, such as chemotherapeutic drugs, nucleic acids, or imaging contrast agents. The hydrophilic nature of polymer coatings enables the efficient loading of hydrophobic drugs via physical encapsulation or chemical conjugation, while the porous structure allows for sustained release kinetics and improved bioavailability [72].

### 4.3. Surface Charge Modification

Surface charge modification is a versatile approach for tuning the physicochemical properties and biological interactions of ZnO nanoparticles. By altering the surface charge through functionalization with charged molecules or polymers, researchers can modulate the cellular uptake, biodistribution, and pharmacokinetics of ZnO nanomaterials, thereby optimizing their therapeutic efficacy and safety profiles [73]. For example, the cationic surface modification of ZnO nanoparticles can promote electrostatic interactions with negatively charged cell membranes, facilitating cellular internalization and intracellular drug delivery. Quaternary ammonium compounds, such as polyethyleneimine (PEI) or chitosan, can be coated onto the surface of ZnO nanoparticles to impart a positive surface charge and enhance their cellular uptake efficiency [74]. Conversely, the anionic surface modification of ZnO nanoparticles can improve their colloidal stability, reduce nonspecific protein adsorption, and mitigate cytotoxicity [75]. Anionic polymers, such as poly(acrylic acid) (PAA) or poly(sodium 4-styrenesulfonate) (PSS), can be adsorbed onto the surface of ZnO nanoparticles, conferring a negative surface charge and improving their biocompatibility under physiological conditions [76].

### 4.4. Bioconjugation Techniques

Bioconjugation techniques, such as click chemistry, thiol chemistry, or carbodiimide-mediated coupling, offer precise control over the functionalization of ZnO nanoparticles with biomolecules, including peptides, proteins, nucleic acids, and imaging probes. These techniques enable the site-specific attachment of biomolecules onto the surface of ZnO nanoparticles, preserving their structural integrity and biological activity [77]. Click chemistry, for instance, involves the selective reaction between azide and alkyne functional groups to form covalent bonds under mild physiological conditions. Azide-functionalized biomolecules can be conjugated onto alkyne-modified ZnO nanoparticles via click chemistry, allowing for precise control over the density and orientation of surface-immobilized ligands [78]. Thiol chemistry, on the other hand, exploits the high affinity between thiol (-SH) groups and gold or silver nanoparticles to facilitate thiol-gold or thiol-silver bond formation. Thiol-functionalized biomolecules, such as peptides or antibodies, can be anchored onto gold-coated ZnO nanoparticles through thiol chemistry, enabling the stable and oriented immobilization of targeting ligands for enhanced tumor targeting and imaging [79]. Carbodiimide-mediated coupling entails activating carboxyl (-COOH) groups on biomolecules through carbodiimide reagents, such as *N*-(3-dimethylaminopropyl)-*N*’-ethylcarbodiimide hydrochloride (EDC) and *N*-hydroxysuccinimide (NHS). Activated biomolecules can then be covalently attached to amino (-NH_2_) groups on the surface of ZnO nanoparticles via amide bond formation, allowing for the efficient coupling of targeting ligands or therapeutic agents [80].

Surface functionalization and modification strategies offer versatile approaches for tailoring the properties of ZnO-based nanomaterials for targeted oral cancer therapy [81]. Researchers can improve the specificity, efficacy, and safety of ZnO nanomaterials and reduce off-target effects through several methods, including conjugating targeting ligands, applying biocompatible polymer coatings, modifying surface charge, and using bioconjugation techniques.. These surface engineering approaches hold promise for advancing precision cancer therapy and improving patient outcomes in oral cancer treatment [82].

## 5. The Therapeutic Efficacy of Zinc Oxide-Based Nanomaterials in Oral Cancer

The potential therapeutic effectiveness of ZnO-based nanomaterials in oral cancer shows promise for enhancing precision cancer therapy (Table 3). ZnO nanoparticles possess inherent anticancer properties due to their capability to produce ROS when exposed to light or ultraviolet (UV) radiation, resulting in cytotoxicity induced by oxidative stress in cancer cells. Additionally, ZnO nanoparticles can be functionalized and engineered to enhance their specificity, stability, and biocompatibility for targeted oral cancer therapy [83]. This section explores the therapeutic efficacy of ZnO-based nanomaterials in oral cancer, including their anticancer mechanisms, performance in in vitro and in vivo studies, and clinical potentials..

### 5.1. Anticancer Mechanisms

The anticancer mechanisms of ZnO nanoparticles involve multiple pathways, including ROS generation, mitochondrial dysfunction, apoptosis induction, and cell cycle arrest [90]. Upon irradiation with light or UV radiation, ZnO nanoparticles absorb photons and become photoexcited, resulting in the generation of reactive oxygen species (ROS) like hydroxyl radicals (•OH) and superoxide ions (O2•^−^) via photocatalytic reactions. The buildup of ROS triggers oxidative harm to cellular elements such as lipids, proteins, and DNA, resulting in mitochondrial dysfunction, membrane permeabilization, and ultimately, apoptotic cell death [93]. Furthermore, ZnO nanoparticles can disrupt the intracellular signaling pathways involved in cell proliferation, survival, and metastasis, thereby inhibiting tumor growth and progression. Studies have demonstrated that ZnO nanoparticles downregulate the expression of anti-apoptotic proteins like Bcl-2 while upregulating pro-apoptotic proteins such as Bax and caspases. This process results in mitochondrial membrane depolarization and caspase-mediated apoptosis in cancer cells [94]. Additionally, ZnO nanoparticles can prompt cell cycle arrest at different checkpoints, including G1/S and G2/M phases, by regulating the expression of cell cycle regulatory proteins like cyclins and cyclin-dependent kinases (CDKs). Consequently, this modulation prevents uncontrolled cell proliferation and inhibits tumor growth [95].

Akhtar et al. (2012) investigated the cytotoxic effects of ZnO NPs on human hepatocellular carcinoma (HepG2), human lung adenocarcinoma (A549), and human bronchial epithelial (BEAS-2B) cells, as well as primary rat astrocytes and hepatocytes, and revealed that ZnO NPs selectively killed cancer cells without affecting normal cells. Further studies on HepG2 cells showed that ZnO NPs upregulated both the mRNA and the protein levels of the tumor suppressor gene p53 and the apoptotic gene bax while downregulating the anti-apoptotic gene bcl-2. Additionally, ZnO NPs induced caspase-3 enzyme activity, DNA fragmentation, reactive oxygen species (ROS) generation, and oxidative stress. This study’s findings demonstrate that ZnO NPs likely induce apoptosis in cancer cells via the ROS-mediated p53 pathway, which is common among anticancer drugs [96].

### 5.2. In Vitro Studies

Multiple in vitro investigations have showcased the anticancer potential of ZnO-based nanomaterials against various oral cancer cell lines, encompassing squamous cell carcinoma (SCC) and mucoepidermoid carcinoma (MEC) cells. These studies have documented the dose-dependent cytotoxicity of ZnO nanoparticles against oral cancer cells, marked by reduced cell viability, proliferation, migration, and invasion, alongside heightened apoptosis and cell cycle arrest. Additionally, ZnO nanoparticles have been shown to exhibit selectivity towards cancer cells over normal cells, indicating their potential as targeted therapeutics for oral cancer [97]. The functionalization of ZnO nanoparticles with targeting ligands, such as folate or epidermal growth factor (EGF), further enhances their cytotoxicity and selectivity towards oral cancer cells by facilitating receptor-mediated endocytosis and intracellular drug delivery [98]. Moreover, combination therapies involving ZnO nanoparticles and conventional chemotherapeutic drugs, such as cisplatin or doxorubicin, have demonstrated synergistic effects on oral cancer cell viability, and these findings suggest the potential for combination approaches to surmount drug resistance and enhance treatment outcomes [66].

Wang et al. (2018) conducted a study to investigate the potential therapeutic efficacy of ZnO NPs in the treatment of oral cancer, namely CAL 27 human tongue cancer cells. Their research demonstrates a dose-dependent decrease in cell viability following ZnO NP treatment, which is attributable to ROS production and mitochondrial damage. Surprisingly, they discover the role of PINK1/Parkin-mediated mitophagy in this cytotoxicity, implying a new mechanism for ZnO NP-induced toxicity. These findings open the door for the further exploration of ZnO NPs as a prospective option for oral cancer therapy, warranting future in vivo studies for clinical translation [84].

Marunganathan et al. (2024) found that marine-derived κ-carrageenan-coated zinc oxide nanoparticles (ZnO-CR NPs) can effectively carry drugs and induce apoptosis in oral cancer cells. The study demonstrates that ZnO-CR NPs have synergistic antibacterial activity against dental infections, as well as a dose-dependent anticancer effect on KB cells. Structural analysis reveals ZnO-CR NPs’ remarkable antibacterial and antioxidant activities, indicating a potential for dental applications. Molecular docking analysis reveals the mechanisms behind their antibacterial action. These findings imply that ZnO-CR NPs are unique tools for tackling oral infections and oral cancer, presenting a viable way to tackle antimicrobial resistance [85].

Verma and Singh (2019) investigate the multifunctional characteristics of a ZnO-rGO nanocomposite. This nanocomposite demonstrates promising potential in non-invasive oral cancer detection through an electrochemical immunosensing approach. The synthesized ZnO–rGO nanocomposite exhibited excellent sensitivity and a low detection limit for interleukin-8 (IL8), a biomarker associated with oral cancer. These findings suggest a significant stride toward developing effective diagnostic tools for oral cancer, potentially revolutionizing early detection and treatment outcomes [86].

Shaik et al.’s 2024 study’s focus on oral cancer underscores the pressing need for innovative therapeutic strategies in combating this disease. Oral cancer presents considerable hurdles due to its complicated etiology and limited treatment options. By using ZnO-PIP NPs to target oral cancer cells, the work opens up a viable therapy option. The capacity of ZnO-PIP NPs to cause apoptosis in oral cancer cells via the BCL2/BAX/P53-signaling pathway has enormous potential for suppressing tumor growth and improving patient outcomes. This approach not only addresses the tumor directly but also complements traditional treatments by targeting dental pathogens, potentially contributing to cancer development or progression. However, while these findings are promising, further research, particularly in vivo studies and clinical trials are essential for assessing the safety and efficacy of ZnO-PIP NPs for potential clinical applications in oral cancer therapy [87].

Tayyeb et al. (2024) investigate the manufacturing and therapeutic potential of curcumin-mediated zinc oxide nanoparticles (ZnO-CU NPs) for oral health, specifically in the treatment of oral cancer. The study provides a multifunctional strategy for tackling oral health concerns by combining zinc oxide nanoparticles’ intrinsic antibacterial characteristics with curcumin’s anticancer effects. The comprehensive characterization of ZnO-CU NPs confirms their structural integrity and functional properties, laying a solid foundation for further investigation. Notably, the study elucidates the complex mechanisms behind ZnO-CU NPs’ antioxidant, antibacterial, and anticancer actions, highlighting their potential as integrated therapeutic agents. While the results are intriguing, more in vivo investigations and clinical trials are required to confirm their safety and efficacy for therapeutic use. This research signifies a significant step towards advancing oral healthcare through innovative nanomedicine approaches, with potential implications for improving treatment outcomes and the quality of life for patients with oral cancer [88].

Shanmugam et al. (2022) present important information about the therapeutic potential of zinc oxide nanoparticles (ZnONPs) in oral cancer treatment. Their study focuses on the prospective anticancer benefits of ZnONPs generated from natural sources. The discussion underscores the importance of apoptosis induction as a key strategy in cancer treatment, emphasizing the unique biochemical and morphological changes associated with apoptotic cell death. The study demonstrates that the formulated CV-ZnONPs effectively triggered apoptosis in oral cancer KB cells through multiple pathways, including ROS accumulation, mitochondrial membrane potential disruption, and the activation of caspases. These findings underscore the potential of CV-ZnONPs as a novel anticancer agent against oral cancer, offering a promising avenue for future research and their development in cancer therapeutics [89].

Wang et al. (2020) investigated the processes driving ZnO-NP-induced apoptosis in GSCC cells, focusing on mitochondrial dysfunction and the p70S6K-signaling pathway. The results indicate that ZnO-NPs have selective cytotoxicity against GSCC cells while sparing normal cells, making them a promising candidate for oral cancer therapy. This comprehensive analysis provides vital insights into the potential of ZnO-NPs as a novel anti-tumor agent for treating GSCC [90].

The research by Ravikumar et al. sheds light on the promising potential of zinc oxide nanoparticles (ZnO NPs) functionalized with cinnamic acid (CA) for addressing various challenges in oral health, particularly in combating oral cancer and dental pathogens. By combining the unique properties of ZnO NPs with the therapeutic attributes of CA, the synthesized ZnO-CA NPs exhibit remarkable antioxidant, antimicrobial, and anticancer activities. The synergistic effects observed underscore the importance of hybrid approaches in nanoparticle-based therapies. Moreover, the molecular docking results provide valuable insights into the mechanisms underlying the interaction between CA and dental pathogen receptors, paving the way for targeted therapeutic interventions. This research signifies a significant step towards the development of innovative dental treatments with broad-spectrum efficacy and tailored applications [91].

The study presents a compelling approach to combat Candida albicans, a major cause of oral candidiasis, using green-synthesized zinc nanoparticles (ZnNPs) derived from Lavandula angustifolia extract. By combining ZnNPs with nystatin, a potent antifungal drug, synergistic effects were observed, enhancing the inhibition and mortality of C. albicans. The study underscores the potential of using nanotechnology for augmenting traditional antifungal therapies, offering a promising avenue for combating oral infections. Additionally, the selective cytotoxicity of ZnNPs towards cancerous cells highlights their therapeutic potential in oral cancer treatment, warranting further investigation into their mechanisms and toxicity profiles [92].

### 5.3. In Vivo Studies

In vivo studies have provided further evidence of the therapeutic efficacy of ZnO-based nanomaterials in oral cancer using animal models, such as xenograft or orthotopic tumor models. ZnO nanoparticles have been shown to inhibit tumor growth, reduce tumor volumes and weights, and prolong survival in oral cancer-bearing mice following systemic or local administration. The histological analysis of tumor tissues has revealed extensive apoptosis, reduced proliferation, and disrupted angiogenesis in response to ZnO nanoparticle treatment, indicating their potential as effective therapeutics for oral cancer [12]. Furthermore, ZnO nanoparticles have good pharmacokinetic and biodistribution patterns in vivo, with their preferential accumulation in tumor tissues due to the increased permeability and retention (EPR) effect. Surface functionalization with targeting ligands increases tumor selectivity and accumulation while reducing off-target effects and systemic toxicity. Additionally, ZnO nanoparticles have been shown to induce minimal acute or chronic toxicity in vital organs, such as the liver, kidneys, and lungs, highlighting their biocompatibility and safety for systemic administration [99].

### 5.4. Clinical Potential

The therapeutic efficacy of ZnO-based nanomaterials in oral cancer holds significant clinical potential for improving patient outcomes and overcoming challenges associated with conventional treatment modalities. Nanotechnology-based approaches offer several advantages over traditional therapies, including enhanced tumor targeting, reduced systemic toxicity, and improved patient compliance. The clinical translation of ZnO-based nanomaterials for oral cancer therapy requires rigorous preclinical evaluation, optimization of formulation and delivery strategies, and compliance with regulatory guidelines for safety and efficacy [100]. Furthermore, the development of multifunctional ZnO-based nanoplatforms, capable of simultaneous imaging, drug delivery, and therapeutic monitoring, holds promise for personalized and precise cancer therapy. Incorporating imaging modalities like fluorescence, magnetic resonance imaging (MRI), or positron emission tomography (PET) facilitates the real-time visualization of tumor growth, metastasis, and treatment response, facilitating early diagnosis and personalized treatment planning [64].

In the study conducted by Vishal et al. (2021), the efficacy of zinc oxide (ZnO) eugenol paste as a dressing material for raw wounds following wide excision of oral potentially malignant disorders (PMDs) is investigated. The combination of zinc’s wound healing properties and eugenol’s antiseptic effects presents a promising option. The results indicate good hemostasis, pain relief, granulation, and epithelialization, with excellent adherence to the wound. Notably, no allergic reactions were observed. Although it is not a replacement for skin grafts, ZnO eugenol paste demonstrates utility as a cost-effective, easy-to-use dressing material for oral surgical wounds, particularly in resource-limited settings. Further research on a larger scale could provide valuable insights [101].

ZnO-based nanomaterials represent a promising therapeutic approach for targeted oral cancer therapy, offering multifaceted anticancer mechanisms, selective cytotoxicity towards cancer cells, and favorable pharmacokinetics and safety profiles in preclinical studies. Further research is warranted to elucidate the molecular mechanisms underlying the therapeutic efficacy of ZnO nanoparticles, optimize formulation and delivery strategies, and translate preclinical findings into clinical applications for the benefit of patients with oral cancer [64].

## 6. Challenges in the Fabrication of ZnO-Based Nanomaterials for Oral Cancer Therapy

While ZnO-based nanomaterials hold great promise for targeted oral cancer therapy, several challenges need to be addressed to realize their full potential. Additionally, exploring future perspectives and research directions is essential for advancing the development and clinical translation of ZnO-based nanotherapeutics [11]. Biocompatibility and Toxicity: Despite their biocompatibility in many preclinical studies, there are still concerns regarding the long-term biocompatibility and potential toxicity of ZnO nanoparticles, particularly upon systemic administration. The accurate assessment of the biocompatibility profile, including acute and chronic toxicity, immunogenicity, and biodistribution, is essential for ensuring the safety of ZnO-based nanotherapeutics in clinical settings [102]. Drug Loading and Release: The efficient loading and controlled release of therapeutic agents from ZnO nanoparticles pose significant challenges due to their high surface area and reactivity. Strategies for improving the drug-loading capacity, stability, and release kinetics while minimizing premature drug leakage and burst release need to be developed for effective drug delivery and sustained therapeutic efficacy [103]. Specificity and Targeting: Achieving the selective targeting and accumulation of ZnO nanoparticles within oral cancer tissues while minimizing off-target effects remains a major challenge. Strategies for enhancing tumor specificity, such as surface functionalizations with targeting ligands or stimuli-responsive moieties, need to be optimized to overcome biological barriers and improve tumor penetration and retention [104]. Regulatory Approval: The regulatory approval and clinical translation of ZnO-based nanotherapeutics for oral cancer therapy require comprehensive preclinical evaluation, including pharmacokinetic studies, toxicity assessments, and efficacy validation in relevant animal models. Meeting regulatory requirements for safety, efficacy, and manufacturing standards is essential for advancing ZnO-based nanotherapeutics from the bench to the bedside [105].

## 7. Future Perspectives

The development of multifunctional ZnO-based nanoplatforms capable of simultaneous imaging, drug delivery, and therapeutic monitoring holds promise for personalized and precision cancer therapy. The integration of imaging modalities, such as fluorescence, MRI or PET, enables the real-time visualization of tumor growth, metastasis, and treatment response, facilitating early diagnosis and treatment optimization [14]. Combining ZnO-based nanotherapeutics with conventional chemotherapy, radiotherapy, or immunotherapy offers synergistic effects and enhanced therapeutic outcomes in oral cancer treatment. Combination therapies can overcome drug resistance, minimize systemic toxicity, and improve treatment efficacy through complementary mechanisms of action, such as ROS generation, DNA damage, or immune modulation [106]. Advancements in targeted drug delivery systems, such as ligand-mediated targeting, stimuli-responsive release, and nanocarrier-based delivery, offer opportunities for enhancing the specificity, efficacy, and safety of ZnO-based nanotherapeutics in oral cancer therapy. The rational design of targeted delivery systems tailored to the unique biological characteristics of oral cancer tissues can improve tumor penetration, cellular uptake, and the therapeutic index while minimizing off-target effects [107]. Theranostic approaches combining therapeutic and diagnostic capabilities within a single nanoplatform enable the real-time monitoring of treatment response and disease progression, facilitating personalized treatment planning and optimization. The integration of imaging probes, biomarkers, and therapeutic agents into ZnO-based nanotherapeutics enables noninvasive imaging, targeted therapy, and therapeutic monitoring, paving the way for personalized and precise cancer therapy [108]. Biomimetic nanomaterials inspired by natural systems offer unique advantages for oral cancer therapy, including enhanced biocompatibility, targeting specificity, and therapeutic efficacy. Mimicking biological structures, functions, and interactions, such as cell membranes, extracellular vesicles, or tumor microenvironments, enables precise control over nanomaterial properties and behaviors, facilitating improved tumor targeting, penetration, and therapeutic outcomes [109]. Addressing the key challenges and exploring future perspectives in the field of ZnO-based nanomaterials for oral cancer therapy is essential for advancing the development and clinical translation of nanotherapeutics. By overcoming technical hurdles, optimizing formulation and delivery strategies, and harnessing innovative approaches, ZnO-based nanotherapeutics hold great promise for improving patient outcomes and revolutionizing the treatment of oral cancer in the future.

## 8. Conclusions

The development of ZnO-based nanomaterials shows great potential for advancing precise cancer therapy, particularly in treating oral cancer. With their unique properties and versatile surface engineering strategies, ZnO-based nanotherapeutics offer a multifunctional platform for targeted drug delivery and imaging. Synthesis methods like sol–gel and hydrothermal synthesis enable precise control over nanoparticle properties, while surface functionalization enhances the specificity and stability for oral cancer treatment. In vitro and in vivo studies demonstrate ZnO-based nanomaterials’ ability to induce cytotoxicity and apoptosis in oral cancer cells with minimal off-target effects. However, challenges such as biocompatibility and regulatory approval remain. Future directions include developing multifunctional nanoplatforms and combination therapies to improve treatment outcomes. In conclusion, ZnO-based nanotherapeutics represent a promising avenue for personalized and effective oral cancer treatment. Collaborative efforts are crucial for translating these advancements into clinical practice, potentially revolutionizing cancer management and saving lives.

## Figures and Tables

**Table 1:** Unique insights into and a comparison of different synthesis methods of ZnO NPs.

S. No	Synthesis Methods		Advantages	Limitations
1.	Physical Synthesis Methods	Chemical Vapor Deposition	High control over film thickness, morphology, and crystallinity; suitable for large-scale production.	Requires high temperatures and sophisticated equipment; relatively high cost.
		Physical Vapor Deposition	Can deposit thin films without chemical reactions; versatile and precise.	Often requires vacuum conditions; may involve high-energy processes.
		Pulsed Laser Deposition	High purity and control over film composition; suitable for complex structures.	Expensive equipment; may have low deposition rates.
		Electrodeposition	Low-cost and scalable; allows fine control over nanostructure growth.	Limited to conductive substrates; may produce uneven films.
		Molecular Beam Epitaxy	Atomic-level control over layer composition; produces high-quality epitaxial films.	Extremely expensive and complex; low throughput.
		Sputtering	High deposition rates; good for uniform thin films.	Requires vacuum and high-energy ions; potential for target contamination.
		Evaporation	Simple and straightforward; effective for thin films.	Limited to materials that can be evaporated; control over thickness can be challenging.
		Magnetron Sputtering	Enhanced ionization efficiency; better control over film properties.	More complex setup than regular sputtering; still requires a vacuum.
		Physical Mixing and Milling	Simple and cost-effective; good for producing nanoparticles.	Limited control over particle size and shape; potential contamination.
		Gas Phase Synthesis	Precise control over nanoparticle size; scalable for large production.	Requires specialized equipment; can be energy-intensive.
2.	Chemical Synthesis Methods	Sol–Gel Method	Versatile; allows fine control over nanoparticle size and distribution.	Requires careful control of reaction conditions; potential for organic solvent use.
		Hydrothermal/ Solvothermal Synthesis	High-quality nanostructures; precise control over shape and size.	High pressure and temperature conditions; longer reaction times.
		Precipitation Method	Simple and cost-effective; scalable.	Limited control over particle size and morphology; potential for agglomeration.
		Microemulsion Method	Good control over particle size and uniformity; mild reaction conditions.	Use of surfactants and organic solvents; may require complex purification.
		Combustion Synthesis	Fast and energy-efficient; high-purity products.	Exothermic reactions can be difficult to control; potential safety hazards.
		Template-Assisted Methods	Allows precise control over nanostructure shape and size.	Removal of templates can be challenging; potential for template contamination.
		Microwave-Assisted Synthesis	Rapid heating; reduced reaction times and energy consumption.	Requires microwave-specific equipment; uneven heating can occur.
		Complex Precursor Decomposition	Controlled release of ZnO; precise control over particle properties.	Complex synthesis and purification steps; potential for by-product formation.
		Spray Pyrolysis	Suitable for large-scale production; uniform thin films.	Requires high-temperature substrates; control over particle size can be difficult.
3.	Biological Synthesis Methods	Plant-Mediated Synthesis	Eco-friendly and sustainable; uses natural reducing agents.	Variability in plant extract composition; scalability can be challenging.
		Microbial Synthesis	Green synthesis route; utilizes natural biological processes.	Requires careful control of microbial culture conditions; slower reaction times.
		Algal-Mediated Synthesis	Utilizes bioactive compounds from algae; environmentally friendly.	Limited control over particle size and shape; scalability issues.
		Enzyme-Assisted Synthesis	High specificity and mild reaction conditions; eco-friendly.	High cost of enzymes; potential for enzyme denaturation.
		Cell-Free Extracts	Uses a rich mixture of biomolecules; green synthesis.	Variability in extract composition; reproducibility can be challenging.
		Biogenic Precipitation	Mimics natural mineralization processes; sustainable.	Slow process; limited control over particle properties.
		Extracellular Synthesis	Simple and eco-friendly; avoids cell lysis.	Lower yields; potential for extracellular contamination.
		Intracellular Synthesis	Protects nanoparticles from aggregation; precise control within cells.	Requires cell lysis for extraction; complex purification needed.
		Biocomposite Formation	Enhanced properties for specific applications; eco-friendly.	Complex synthesis; limited scalability.
		Green Synthesis Approaches	Low toxicity and biocompatibility; environmentally sustainable.	Often slower and less efficient; may have lower yields.

**Table 2:** Other properties and a comparison and analysis of different synthesis methods of ZnO NPs.

S. No	Comparison and Analysis	Physical Methods	Chemical Methods	Biological Methods
1.	Control Over Nanostructure Properties	Physical methods generally offer precise control over size, morphology, and crystallinity but require sophisticated equipment and conditions.	Chemical methods provide versatility and scalability but often involve hazardous chemicals.	Biological methods are environmentally friendly and sustainable but can be less controllable and slower.
2.	Scalability	They are more easily scalable for industrial production.	They are more easily scalable for industrial production.	Biological methods face challenges in scaling up due to their variability in biological materials.
3.	Environmental Impact	Physical methods can be energy-intensive.	Chemical methods often involve toxic reagents and solvents.	Biological methods are the most environmentally friendly, using natural agents and mild conditions.
4.	Cost	Chemical methods vary widely in cost depending on the specific process and reagents used.	Physical methods often involve high initial capital investments in equipment.	Biological methods are typically less expensive due to the use of natural and renewable resources, but the complexity of control can increase costs.
5.	Application Suitability	Physical methods are suitable for high-tech applications requiring precise control, such as electronics and optoelectronics.	Chemical methods are versatile and suitable for a wide range of applications, including catalysis and sensing.	Biological methods are particularly suitable for biomedical and environmental applications due to their biocompatibility and eco-friendliness.

**Table 3:** Zinc oxide (ZnO)-based nanomaterials in oral cancer hold promise for advancing precise cancer therapy.

S. No.	Materials	Size	Preparation Method	Characterization Methods	Biological Studies (In Vitro/In Vivo)	Key Findings in Oral Cancer	References
1.	ZnO nanoparticles	~50 nm	Not specified	TEM, XRD, DLS	In vitro	Promoting toxicity in CAL 27 oral cancer cell lines via the activation of PINK1/Parkin-mediated mitophagy.	[84]
2.	ZnO-CR Nanoparticles	100–200 nm	Green synthesis method	SEM, FTIR, XRD	In vitro	Induced apoptosis in KB cells.	[85]
3.	ZnO—reduced graphene oxide (ZnO–rGO) nanocomposite	3–5 nm	Sol–gel process	Powder XRD, UV–Vis spectroscopy, EDX, TEM	In vitro	The ZnO–rGO nanocomposite exhibited high sensitivity and a low detection limit for IL8, demonstrating its potential for non-invasive oral cancer detection.	[86]
4.	ZnO—Piperine Nanoparticles (ZnO-PIP NPs)	50–100 nm	Green synthesis	UV spectroscopy, SEM, XRD, FTIR, EDAX	In vitro	Induction of apoptosis in oral cancer cells via the BCL2/BAX/P53 signaling pathway.	[87]
5.	ZnO-CU Nanoparticles (Curcumin-mediated ZnO NPs)	100–200 nm	Green synthesis	SEM, EDAX, UV spectroscopy, FTIR, XRD	In vitro	Induced apoptosis in oral cancer cells through the BCL2/BAX/P53 pathway.	[88]
6.	ZnO Nanoparticles (CV-ZnONPs)	30–45 nm	Green synthesis	UV–Vis spectrophotometry, XRD, TEM, EDX, FT-IR, PL	In vitro	Inhibited KB cell viability, decreased MMP, enhanced ROS levels, induced apoptotic cell death, altered nuclear morphology, increased caspase activity.	[89]
7.	ZnO Nanoparticles	50–100 nm	Synthesized using a precipitation method	TEM, XRD, FTIR	In vitro	ZnO-NPs induced growth inhibition and apoptosis in gingival squamous cell carcinoma (GSCC) cells but not in normal cells.	[90]
8.	ZnO-CA Nanoparticles	100–200 nm	Chemical synthesis	SEM, FTIR, XRD	In vitro	ZnO-CA NPs exhibit dose-dependent anticancer activity against Human Oral Epidermal Carcinoma KB cells.	[91]
9.	ZnO Nanoparticles	30–80 nm	Green synthesis	UV-VIS spectral analysis, EDX analysis, XRD pattern analysis, FTIR analysis, SEM analysis	In vitro	Potent antifungal effect against Candida albicans, synergistic effect with nystatin, cytotoxic to oral cancer cells but non-toxic to normal cells.	[92]

## Data Availability

No new data were created.
